# Assessment of cardiovascular risk factors among HIV-infected patients aged 50 years and older in Cameroon

**DOI:** 10.3934/publichealth.2022034

**Published:** 2022-06-08

**Authors:** Henri Olivier Tatsilong Pambou, Amandine Gagneux-Brunon, Bertrand Tatsinkou Fossi, Frederic Roche, Jessica Guyot, Elisabeth Botelho-Nevers, Caroline Dupre, Bienvenu Bongue, Celine Nguefeu Nkenfou

**Affiliations:** 1 University of Buea, Faculty of Sciences, Buea, Cameroon; 2 INSERM, UMR 1059, SAINBIOSE, University Jean Monnet, Saint-Etienne, France; 3 CIRI-Centre International de Recherche en Infectiologie, Team GIMAP, Univ Lyon, Université Jean Monnet, Inserm, U1111, CNRS, UMR530, F42023 Saint-Etienne, France; 4 Chantal Biya International Reference Centre, System Biology Laboratory, Yaoundé, Cameroon; 5 University of Yaoundé I, Higher Normal School of Yaoundé, Yaoundé Cameroon

**Keywords:** HDL cholesterol, risk of cardiovascular events, PLHIV

## Abstract

**Background:**

Increasing the longevity of people living with HIV (PLHIV) around the world has been accompanied by an increase in the prevalence of cardiovascular disease (CVD) risk factors and morbidity. The impact of these trends on the epidemiology of CVD among PLHIV is less clear. The aim of this study was to assess the risk factors for CVD, and to estimate these risks at 10 years in PLHIV aged 50 and above.

**Methods:**

This was a descriptive and analytical study carried out at Mvog Ada District Hospital in Yaounde, Cameroon from January 2020 to January 2021. Descriptive bivariate analyses were used to present the data. The data are presented as frequencies and percentages for categorical variables, and in terms of means and standard deviations for continuous variables where appropriate. The 10-year CVD risk score was calculated using two tools: the validated Framingham risk score (FRS) (low < 10%, moderate 10–20% and high ≥ 20%) and SCORE score (SSC) (low < 3%, moderate 3–4% and high ≥ 5%). Multiple logistic regression models were constructed to examine the respective relationships between the binary dependent variable high CVD risk (FRS ≥ 20%) and the population group, alcohol consumption (more than 10 glasses of beer per week, or more than 35.7 cl/day) and hypertriglyceridemia (independent variables). A p-value less than or equal to 0.05 was considered statistically significant.

**Results:**

A total of 112 people aged 50 and above were enrolled in the study out of 180 people registered at the HIV care unit, that is a participation rate of 62.22%. The average age of the participants was 57.3 ± 6.4 years, and the female/male ratio was 1.6. The majority of participants (53.57%) had normal glycaemia levels (<1.10 g/L), 4.46% were diabetic and 46.40% had high blood pressure. The adherence rate for ARV treatment was 98.20%; most participants (77.20%) were alcohol consumers, and 28.10% of participants had hypertriglyceridemia. The estimates of overall cardiovascular risk in 10 years presented 50.90% of participants with low risk, 33% with moderate risk and 16.10% with high risk.

**Conclusions:**

Our study indicated an overall risk of cardiovascular events in 10 years is 16.10%, with the main conditional risk factor being hypertriglyceridemia and alcohol consumption, which appeared to triple the risk of CVD among PLHIV.

## 1. Introduction

In 2021, UNAIDS estimated that there were 37.7 million people living with HIV (PLHIV) worldwide, with nearly 27.5 million having access to antiretroviral (ARV) treatment [1]. Since the introduction of ARVs in the 1990s, the life expectancy of PLHIV has increased steadily, particularly, with an increase of older PLHIV. In 2013, UNAIDS highlighted the increase in the number of PLHIV over the age of 50 and estimated that there are 3.6 million PLHIV over the age of 50 worldwide [2], with almost 30% of PLHIV over 50 years of age in developed countries. In resource-limited countries, at the beginning of the epidemic, about 10% of PLHIV were over 50 years of age; however, by 2030, nearly 73% of patients will be over 50 compared to 28% in 2010. Additionally, although 29% of HIV-infected patients had a non-infectious comorbidity in 2010, it is estimated that this proportion will be 84% in 2030 [3],[4]. Aging of the PLHIV population is accompanied by challenges, that is the management of comorbidities, particularly, cardiovascular comorbidities, the consequences of multiple medications [3],[5] and the impact on the quality of life. However, despite progress in the management and care of PLHIV, their life expectancy seems to remain lower than that of the general population, even in high-resource countries such as the USA [6],[7]. Many factors appear to be associated with cardiovascular risk. Death from cardiovascular disease (CVD) is now a major health threat to PLHIV, reflecting trends seen in the general population. Emerging data suggests that HIV-infected patients in sub-Saharan Africa (SSA) face the same increased burden of CVD. Unfortunately, in SSA, CVD risk reduction often does not receive special attention in the primary care of PLHIV. However, identifying HIV-infected patients who are at increased risk, as well as candidates for the primary prevention of CVD risk, remains to be challenging. Among the preventive measures for CVD, the monitoring of dyslipidaemia, high blood pressure and obesity appear to be effective. Thus, several risk prediction models developed based on the general population are available to predict CVD risk [8], the most notable being the USA-based pooled cohort equations [9], the Framingham risk functions, and the Europe-based systematic coronary risk evaluation (SCORE) [10]. In validation studies done in cohorts of PLHIV, these models generally underestimate CVD risk, especially in individuals who are younger, female, racially Black, or predicted to be at low/intermediate risk. An HIV-specific CVD prediction model, the Data Collection on Adverse Events of Anti-HIV Drugs (DAD) model, is available, but its performance is modest, especially in USA-based cohorts [11]. Enhancing CVD prediction with novel biomarkers of inflammation or coronary artery calcification is of interest, but has not yet been evaluated in PLHIV. Lastly, studies on CVD risk prediction are lacking in diverse PLHIV globally, the use of the Framingham equation or other developed tools exclusively for populations with HIV infection such as the Data collection on Adverse effects of anti-HIV Drugs (DAD) would be essential in the routine follow-up [12]. Our objective was to assess the risk factors for CVD among PLHIV+50, and to estimate the risk of cardiovascular events in 10 years using two different tools to enable the development of preventive measures.

## 2. Materials and methods

### 2.1. Type, location and study population

This was a cross-sectional study carried out from March 2020 to January 2021 at the HIV care unit of the Mvog-Ada district medical centre, which is part of the Djoungolo health district, one of the eight health districts of Yaoundé, the capital of Cameroon. This HIV care unit has 1300 patients actively in care, with about 18% of the patients aged 50 or over. The participants who had come for their routine visit during the study period were recruited regardless of their treatment protocol, gender, or duration of treatment, and those who voluntarily agreed to participate in the study. The study participants did not include HIV-negative persons or those under 50 years of age. Those participants with incomplete data were excluded. A total of 112 participants consented and were enrolled in our study (see the flow chart for participant enrollment in Supplementary Data).

### 2.2. Data collection and laboratory analyses

The participants were invited to the counselling sites of the health centre for enrollment into the study. A standardised case record form was used to collect socio-demographic information (i.e., age, sex and marital status), self-reported lifestyle factors (i.e., smoking, alcohol consumption and physical activity), self-reported health status (i.e., diagnosis and treatment for hypertension, heart disease and diabetes). In addition, anthropometric parameters (i.e., weight and height) were measured for each participant. Data on polymedication, clinical and biological parameters (i.e., blood pressure, body mass, blood glucose, total cholesterol (TC), low-density lipoprotein cholesterol (LDL-C), high-density lipoprotein cholesterol (HDL-C) and triglycerides) were recorded for each participant.

### 2.3. Clinical parameters

Blood pressure was measured using a European Society of Hypertension-validated Health ease digital blood pressure (BP) instrument. Three readings of systolic and diastolic BP were taken from a seated and rested participant; there was at least 5-min rest interval between each reading. The average of the three readings was used to classify participants as normal (systolic BP (SBP) < 120 mm Hg or diastolic BP (DBP) < 80 mm Hg), pre-hypertensive (SBP 120–139 mm Hg or DBP 80–89 mm Hg) or hypertensive (SBP ≥ 140 mm Hg or DBP ≥ 90 mm Hg).

The body mass index (BMI) for each participant was calculated from the weight and height measurements as weight in kilograms divided by height in square meters. Regarding the variable “obesity”, the BMI was categorised as normal (BMI ≤ 24.90), overweight (BMI 25–29.90) or obese (BMI ≥ 30).

We assessed the level of physical activity and sedentary lifestyle using the Ricci and Gagnon questionnaire. The Ricci and Gagnon questionnaire is a self-assessment questionnaire that classifies the physical activity profile as inactive, active or very active; it is calculated by adding the number of points (1 to 5) corresponding to the box ticked for each question. It was initially presented by J. Ricci and L. Gagnon [13] of the University of Montreal before being modified by F. Laureyns and JM. Séné in 2016 [https://www.ameli.fr/sites/default/files/questionnaire-activite-physique_cpam-haute-savoie.pdf; accessed on 24 June 2021].

### 2.4. Biological parameters

The lipid profile for each participant was assessed from the venous blood specimen. The total cholesterol was quantified by using a colorimetric method (Cholesterol liquicolor, Universal reagent; cat n° 10017, HUMAN, Max-Planck-Ring 21, 65205 Wiesbaden, Germany); the optical density (OD) was read using the HUMALYSER spectrophotometer (Humalyzer 3500 HUMAN) and interpreted as follows: <190 mg/dl = normal and >190 mg/dl = high). Triglycerides quantities were determined by following the same procedure as that for the total cholesterol using the Triglycerides liquicolor, Universal reagent, cat n° 10724 (HUMAN, Max-Planck-Ring 21, 65205 Wiesbaden, Germany); it was interpreted as follows: normal: <150 mg/dl, slightly elevated: 150–200 mg/dl, high 200–500 mg/dl or very high ≥500 mg/dl. The HDL-C was quantified by employing an enzymatic colorimetric test (liquicolor, Universal reagent). The HDL-C presents a high diagnostic value for the assessment of the individual risk of developing coronary heart disease. Its interpretation is gender-specific, as follows: men (>55 mg/dl = no risk; 35–55 mg/dl = relative risk; <35 mg/dl = high risk); women (>65 mg/dl = no risk; 45–65 mg/dl = relative risk, <45 mg/dl = high risk). The LDL-C was deduced using the Friedewald formula, as follows: LDL-C = TC − [HDL-C + Tg/5] (in g/L); a normal value is less than 1.60 g/L in men and 1.50 g/L in women.

Blood glucose dosage was determined by using a ONE TOUCH VIERA glucometer; the interpretation is as follows: less than 0.70 g/L of blood = hypoglycaemia; between 0.70 and 1 g/L of blood = normal blood glucose; between 1 and 1.25 g/L of blood = moderate hyperglycaemia; greater than 1.26 g/L of blood = diabetes.

### 2.5. Cardiovascular risk assessment

Calculations of the cardiovascular risk in 10 years were performed by using the most commonly used cardiovascular risk calculator (www.cardiorisk.fr). The equations for these “risk functions” are based on the Cox statistical, which includes parameters such as age, sex, total cholesterol, HDL-C, SBP, tobacco consumption, known status of diabetes and hypertension. The 10-year cardiovascular risk was estimated by applying two formulas for each subject, who was classified as being at low, moderate or high risk. These are the Framingham formula and SCORE formula. The Framingham model is used by the NIH (USA), and was validated after a vast program of permanent research (since 1948). D'Agostino confirmed it in 2008 following the works of Anderson 1991 and Wilson 1998 [14],[15]. The SCORE formula, which is used by the ESC (Europe), was validated by a series of studies that were carried out in 12 European countries in 2003 by Conroy [16].

Patients with established coronary heart disease or another atherosclerotic disease(s) were directly defined as having high cardiovascular risk (>20%) [14]–[16].

### 2.6. Ethical considerations

Written informed consent was obtained from all participants, and participant confidentiality was respected throughout the study. The study was approved by the Human Health Research Council Ethics Committee of Centre Regional Cameroon (CE n°. 2131/CRERSHC/2020).

### 2.7. Data analysis

The data were parsed using descriptive bivariate analysis. The data were presented as frequencies and percentages for categorical variables, and as means ± standard deviations for continuous variables where appropriate. Multiple logistic regression models were constructed to examine the respective relationships between the binary dependent variable, high CVD risk (Framingham Risk Score (FRS) ≥ 20%), and physical activity, alcohol use, obesity and triglycerides (independent variables). These factors are known from the literature to be commonly associated with cardio-metabolic morbidity. Some of the factors used to determine the Framingham formula (FRS) outcome, such as age, hypertension and total cholesterol, were, however, excluded from the models. A p-value and two-tailed p-value of <0.05 were considered to be statistically significant. All analyses were done using IBM SPSS software (version 20, Chicago, Illinois, USA). The data were codified before analysis. Age was grouped into four categories: 50–55, 56–60, 61–65 and over 65. Occupation was grouped as follows: “worker” for participants with an occupation (e.g., bricklayer, farmer, dressmaker, civil servant, shopkeeper or driver), and “unemployed” for those who did not leave their homes regularly (e.g., housewife or retired). Physical activity was classified as “active” for participants practising a sport (e.g., walking, running and/or cycling), or “inactive” for sedentary participants (i.e., those spending more than 7 hours continuously seated). Cardiovascular risk was estimated for each subject by the two risk equations, and subjects were then classified as having a low, moderate or high 10-year coronary risk by using the Framingham equation, (<10%, 10–20% and ≥20%, respectively) and SCORE equation (<3%, 3–4% and ≥5%, respectively).

## 3. Results

### 3.1. Demographic and clinical characteristics of study participants

A total of 112 PLHIV aged 50 years or over were enrolled in the study. Their age ranged from 50 to 77 years, with an average age of 57.3 ± 6.7 years. Women (62.50%) were more represented than men (37.5%). The most represented age range was between 50 and 55 years, with a percentage of 43.60%. The majority of participants were employed (i.e., workers) (60.70%). We had 33.9% overweight participants, and 18.80% obese participants; additionally, 46.60% had high blood pressure. Nearly half of the participants did not engage in any physical activity (i.e., classified as inactive) (49.10%) and 50.90% declared to often take a walk, run or cycle at times (Table 1).

**Table 1.** Demographic characteristics of study participants.

Variable	Number	Percentage (%)
**Age (years)**
50–55	**49**	**43.80**
56–60	27	24.10
61–65	23	20.50
66 and over	13	11.60
**Sex**
Female	**70**	**62.50**
Male	42	37.50
**Marital status**
Single	23	20.50
Married	59	52.70
Widow/widower	30	26.80
**Job status**
Employed	**68**	**60.70**
Unemployed	44	39.30
**BMI**
Normal	53	47.30
Overweight	**38**	**33.90**
Obese	21	18.80
**Blood pressure**
Low	3	2.70
Normal	57	50.90
High	**52**	**46.40**
**Physical activity**
Active	56	50.90
Inactive	54	49.10
**Alcohol consumption**
No	38	33.90
Yes	**74**	**66.10**
**Tobacco consumption**
No	103	92.00
Yes	9	8.00
**Duration under treatment**
<6 years	63	56.30
≥6 years	49	43.70
**Compliance to ARV treatment**
No	2	1.80
Yes	110	98.20
**Sedentary lifestyle**
No	52	46.40
Yes	60	53.60

### 3.2. BP profile of patients

Fifty-two of 112 participants (46.40%) had high BP (SBP ≥ 140 mm Hg and DBP ≥ 90 mm Hg). Those taking BP medications (5/52) were qualified as hypertensive. Those with normal BP represented 53.60% (60/112) of the study population.

### 3.3. Compliance to ARV treatment of patients

All participants were on first-line therapy (Tenofovir, Lamivudine, and dolutegravir), which is taken as a single tablet daily, with ease of adherence. This was justified by a 92.59% viral load suppression rate among the participants of the study. Adherence was high (98.2%) in our study population. Less than 1% of participants declared to have missed their treatment doses. The high rate of adherence may be explained by the fact that the participants are members of HIV care support groups and are contacted from time to time as part of their follow-up.

### 3.4. Lipid profile of participants

From our results, dyslipidemia was especially observed among women, as they showed total cholesterol (67.85%) and triglycerides (56.52%) levels that were slightly higher than in men. Regarding HDL-C, which is a marker of cardiovascular events, it was found that women aged 50 years and over had low HDL-C levels (84%) compared to men (16%) (p = 0.005).

As for glycaemia, 76.8% of the participants had normal blood glucose levels, 19.6% presented high levels and 3.6% were known to be diabetic. High total cholesterol was found in 25% of the study participants, and high triglyceride was found among 20.50%. Low HDL-C was found in 44.60% of the participants. A total of 5.40% had LDL-C (Table 2).

**Table 2.** Biological characteristics of study participants.

Variable	Number	Percentage (%)
**Blood glucose**
Normal	78	76.80
High	22	19.60
Diabetic	4	3.60
**Total cholesterol**
Normal	84	75.00
High	28	25.00
**Triglyceride**
Normal	89	79.50
High	**23**	**20.50**
**HDL-C**
Low	50	44.60
Intermediate	55	49.10
Normal	7	6.30
**LDL-C**
Abnormal	6	5.40
Normal	106	94.60

### 3.5. Prevalence of CVD risk using FRS and SCORE scores

The estimated FRS and SCORE (SSc) scores of the average 10-year risk of CVD in this sample population was 16.10% and 12.50%, respectively, with the average being significantly higher in men than in women (33.30% vs. 5.70%, and 21.40% vs. 7.10%, respectively) (p < 0.001). Table 3 presents the risk scores according to gender.

**Table 3.** Distribution of the risk scores among participants according to gender.

Variables	Score of Framingham			Score of SCORE (SSc)		
	Female N = 70	male N = 42	Total N = 112	Female N = 70	Male N = 42	Total N = 112
**Low risk**	47(67.20%)	8(19.04%)	55(49.10%)	58(82.90%)	18(42.90%)	75(66.90%)
**Moderate risk**	19(27.10%)	20(47.60%)	39(34.80%)	7(10.00%)	15(35.70%)	22(19.60%)
**High risk**	4(5.70%)	**14(33.30%)**	**18(16.10%)**	5(7.10%)	9(21.40%)	14(12.50%)
**P-value**	<0.00001			<0.00001

### 3.6. Factors associated with cardiovascular events

**Figure 1.**
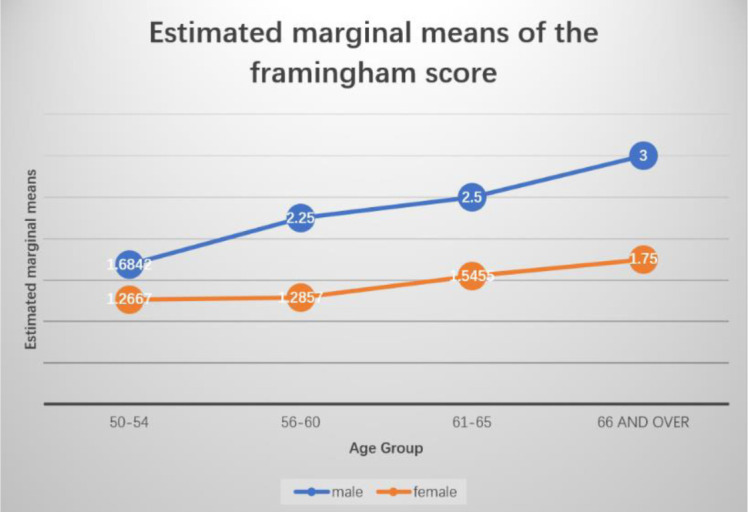
Percentages of male and female participants with an estimated high CVD risk.

The 10-year high CVD risk increased with age, with 38.80% of people aged 56 to 60 being at risk; this is higher than the overall studied population. Although the trends were similar, there was a considerable difference between men and women when it came to the level of CVD risk (men 33.33% vs. women 5.70%, p = 0.00001). The proportion at high risk for CVD increased exponentially overall in men. These data are presented in Figure 1.

### 3.7. Variables associated with cardiovascular risk

Table 4 shows that age, sex, blood pressure, smoking, alcohol consumption and increased blood triglycerides are strongly associated with CVD when the risk is high or moderate.

**Table 4.** Variables associated with high risk or moderate risk of CVD.

Variable	Total		Low		High risk or moderate risk		*P*
	N	%	N	%	N	%	
**Age**
50–55	49	43.80	30	54.50	19	33.30	**0.026**
56–60	33	29.50	16	29.10	17	29.80	
61–65	17	15.20	7	12.70	10	17.50	
66 and over	13	11.60	2	3.60	11	19.30	
**Sex**
Female	70	62.50	47	85.50	23	40.40	**<0.0001**
Male	42	37.50	8	14.50	34	59.60	
**Status**
Single	24	29.30	16	38.10	8	20.00	0.072
Married	58	70.70	26	61.90	32	80.00	
Widow	0	0.00	0	0.00	0	0.00	
**Profession**
Unemployed	44	39.30	21	38.20	23	40.40	0.814
Worker	68	60.70	34	61.80	34	59.60	
**BMI**
Normal	53	47.30	27	49.10	26	45.60	0.084
Overweight	38	33.90	14	25.50	24	42.10	
Obese	21	18.80	14	25.50	7	12.30	
**Blood pressure**	
Low	3	2.70	2	3.60	1	1.80	**<0.0001**
High	52	46.40	12	21.80	40	70.20	
Normal	57	50.90	41	74.50	16	28.10	
**Physical activity**
Active	56	50.00	26	47.30	30	52.60	0.325
Inactive	54	48.20	27	49.10	27	47.40	
**Smoker**
No	103	92.00	54	98.20	49	86.00	**0.011**
Yes	9	8.00	1	1.80	8	14.00	
**Alcohol consumption**
No	38	33.90	25	45.50	13	22.80	**0.017**
Yes	74	66.10	30	54.50	44	77.20	
**Duration**
<6	68	60.70	31	56.40	37	64.90	0.354
≥6	44	39.30	24	43.60	20	35.10	
**Adherence**
No	2	1.80	1	1.80	1	1.80	0.980
Yes	110	98.20	54	98.20	56	98.20	
**Sedentary lifestyle**
No	53	47.30	24	43.60	29	50.90	0.443
Yes	59	52.70	31	56.40	28	49.10	
**Total cholesterol**
Normal	84	75.00	43	78.20	41	71.90	0.445
High	28	25.00	12	21.80	16	28.10	
**HDL-C**
Low	50	44.60	29	52.70	21	36.80	0.166
Intermediate	55	49.10	22	40.00	33	57.90	
Normal	7	6.30	4	7.30	3	5.30	
**LDL-C**
Abnormal	6	5.40	4	7.30	2	3.50	0.376
Normal	106	94.60	51	92.70	55	96.50	
**Triglyceride**
Normal	89	79.50	48	87.30	41	71.90	**0.044**
High	23	20.50	7	12.70	16	28.10	
**Fasting blood sugar**
Normal	90	80.40	45	80.40	45	80.40	0.832
High	22	19.60	11	19.60	11	19.60	

*Note: %: percentage; BMI: body mass index; HDL: High-density lipid; LDL: low-density lipid; *P*: p-value (a p-value less than 0.05 presented in the table in bold is considered significant).

Stepwise regression analysis (Table 5) was used to assess the associations between alcohol consumption and the level of triglyceride and CDV risk.

**Table 5.** Risk influencing factors and stepwise regression analyses.

Variable	N	High or moderate risk	OR (95% CI)	P-value
**Alcohol consumption**				
No	38	13	1	0.022
Yes	74	44	2.62 (1.150–6.011)	
**Triglyceride**				
Normal	89	41	2.40 (0.880–6.547)	0.080
High	23	16	1	
		**High risk**		
**Triglyceride**				
Normal	89	11	1	0.042
High	23	7	3.10 (1.043–9.224)	0.042

*Note: OR: odds ratio; CI: confidence interval.

## 4. Discussion

In this study, using the FRS and SCORE score, our main objectives were to determine the 10-year risk of CVD, and to assess the associated factors in PLHIV+50. The target population was 234 patients, but 180 were excluded; 112 participated, yielding a participation rate of 47.87%. This participation rate is low, and this could be justified by the fact that enrollment was carried out during the COVID-19 period, and most patients prefer to receive their treatment through an intermediate without travelling to hospital.

The use and clinical benefits of CVD risk algorithms (e.g., the FRS and SCORE score) have been widely discussed in recent years, and the question continues as to which algorithm is more appropriate for HIV-infected populations. In our study, the two algorithms presented different 10-year predictions, that is, 12.50% for SCORE and 16.10% for Framingham. The Framingham algorithm seemed a bit more comprehensive because it incorporated more risk factors (e.g., diabetes). However, the prevalence in this study was higher than that found in other studies [17] of HIV-infected adults aged 30–50 years in the Gaborone, Botswana population. The high prevalence of CVD risk (16.1%) calculated using the FRS was much closer to that reported in the Makandwe study in 2021 (17%). Once again, the similarities can be attributed to the fact that both studies were conducted on an older population. However, our results on the prevalence of CVD risk are in accordance with studies conducted in HIV-positive people aged 40 years and older in Taiwan, and in an HIV outpatient clinic in the city of Vitoria, Espirito Santo, Brazil [18],[19]. A local study by Noumegni and colleagues in 2017 [20] on a younger population (mean age: 44) at the Yaoundé Central Hospital showed a cumulative prevalence (high risk and very high risk) of 8.40%, which is lower than that found in the present study. This could be justified by the fact that the risk of CVD increases with age, and that the study was carried out on non-HIV-infected participants.

Age, being a risk factor that reflects the duration of an individual's exposure to other risk factors, is an independent factor for CVD, and should be understood in terms of structural ageing of the heart and vessels leading to their remodelling; this was estimated to be 11.90% in men and 40.30% in women, without a plausible explanation of this sex-related difference [21]. High BP is one of the traditional risk factors for CVD. High BP was reported in 46.40% of the study population, but only 23.07% (12/52) were aware of their hypertensive status and undertreatment; the rest were diagnosed during our study and referred to their physician. High BP is known to genetically predispose African populations to CVD [22].

The distribution of risk according to age group and sex (Figure 1) shows that men are two to three times more likely to suffer from CVD than women, whereas, after the age of 65, the risk of heart disease is about the same for both sexes. We also found that the 10-year risk of CVD was higher for men (33.30%) than for women (5.70%). Our results regarding the higher risk of CVD in men agree with what has been recently reported by Melo and Makandwe [23],[24], who conducted a cross-sectional study that assessed the risk of CVD in PLHIV using the FRS. The higher risk in men could be attributed to smoking and alcohol drinking. Smoking 1 to 4 cigarettes per day was associated with a 3-fold higher risk of dying of a heart attack. In our study, men smoked an average of five sticks of cigarettes per day compared to less than one stick of cigarettes per day for women (OR = 3.7; 95% CI = 0.87–15.77; p = 0.05). Regarding alcohol consumption, men drank an average of 1.3 L of alcohol per day, and women drank 0.5 L of alcohol per day (OR = 2.59; 95% CI = 1.07–6.23; p = 0.03). Thus, these risk factors were found to be significantly associated with CVD events. These findings are in agreement with those of the studies by Gaetano and Wu [18],[25] on HIV-positive people aged 40 years and over in Taiwan.

In terms of employment status, 39.30% of the participants were jobless, and 60.70% had an occupation. Having a small remunerating job seemed to be a significant factor in increasing the risk for CVD, as it provided them some financial power to afford drink.

The data collected using the Ricci and Gagnon questionnaire showed that 50.90% of the participants were active, with the majority regularly walking. A lack of regular physical activity has been associated with an increased risk of cardiovascular mortality in most epidemiological studies [26]. Physical activity is known as a stroke-preventive tool. It is understood that some ARVs can trigger hyperlipidemia, impair glucose tolerance, etc. Almost all of the study participants were on Tenofovir-lamivudine-dolutegravir. The intake of dolutegravir is known to promote weight gain, and can increase the risk of CVD in HIV-positive people [25],[28]. Policarpo et al. (2019) [27] further identified high triglycerides and low HDL-C as key factors in the increased CVD risk in HIV-positive individuals. Indeed, among the participants of our study, high triglyceridemia was found to be associated with high CVD risk (p = 0.04). HIV has also been identified as a contributor to an increased likelihood of cardio-metabolic disorders [27]. Peyracchia and colleagues [29] confirmed that an advanced HIV infection is associated with a high risk of non-calcific plaques and a worse prognosis, including cardiovascular events and Acute Coronary Syndrome recurrence. The risk of CVD may increase with prolonged duration of ARV treatment [30].

In order to study these associations, two models were considered. The first model considered low or moderate risk as compared to high risk. The second model considered low, moderate and high risk separately. Stepwise regression analyses were performed. From the first model (16.10% cardiovascular risk), hypertriglyceridemia was shown to be strongly associated with cardiovascular events (OR = 3.10; 95% CI = 1.043–9.224; p = 0.04), meaning that, in the case of hypertriglyceridemia, the patient has 3.1 times the risk of developing a cardiovascular event. According to the second model (cardiovascular risk of 50.90%), alcohol consumption was found to be associated with cardiovascular events in PLHIV+50 (OR = 2.62; 95% CI = 1.150–6.011; p = 0.02), showing that alcohol drinkers have 2.6 times the risk of developing a cardiovascular event.

Nevertheless, this study presented some limitations. The participants were all from the same urban referral health facilities and were not randomly selected; this may limit the generalisability of our results. Our sample also included a very high proportion of women, which may in some ways bias our results. However, it is generally observed that female participation in surveys tend to be very high in Africa. The tools used for CVD risk calculation are those used for the general population. However, there are tools more specific to PLHIV, such as the DAD protocol, and, especially, the American College of Cardiology/American Heart Association atherosclerotic cardiovascular disease risk estimator published in 2019 by Kelly Young [31]. This second tool takes into account the patient in his globality, including the socio-economic and lifestyle factors. In our study, the patients' histories were unavailable. Despite these limitations, the results obtained are consistent with those reported by Noumegni in 2017 [20]. Our sample size was small compared to other similar studies. However, the distribution analysis was similar to the Makandwe 2021 [23] study in South Africa conducted on a similar population.

Given our findings, we thus propose a sensitisation towards the following for the better management of PLHIV aged over 50 in Cameroon: limiting alcohol consumption, adopting a healthy diet, adopting appropriate levels of physical activity and considering weight loss in order to further reduce the risk of cardiovascular events.

## 5. Conclusions

The trends of ageing and increased cardiovascular risk, especially in PLHIV in developed countries, were found in our study. These results show that an assessment of overall risk based on the Framingham and Score algorithms, and considering the sum of all major risk factors, may be clinically useful. From these results, we suggest periodic evaluations of CVD risk in HIV patients so as 1) to identify high-risk patients, and 2) provide education and sensitisation to this risk group.
